# Three new species of *Exocelina* Broun, 1886 from the southern slopes of the New Guinea central range, with introduction of the *Exocelina skalei* group (Coleoptera, Dytiscidae, Copelatinae)

**DOI:** 10.3897/zookeys.1007.59351

**Published:** 2020-12-30

**Authors:** Helena Shaverdo, Suriani Surbakti, Bob Sumoked, Michael Balke

**Affiliations:** 1 Naturhistorisches Museum Wien, Burgring 7, 1010, Vienna, Austria Naturhistorisches Museum Wien Vienna Austria; 2 Department of Biology, Universitas Cendrawasih, Waena, Papua, Indonesia Universitas Cendrawasih Papua Indonesia; 3 Walian 2, Tomohon Selatan, N Sulawesi 95439, Indonesia Unaffiliated Tomohon Selatan Indonesia; 4 SNSB-Zoologische Staatssammlung München, Münchhausenstraße 21, D-81247, Munich, Germany SNSB-Zoologische Staatssammlung München Munich Germany; 5 GeoBioCenter, Ludwig-Maximilians-University, Munich, Germany GeoBioCenter, Ludwig-Maximilians-University Munich Germany

**Keywords:** Australasia, distribution, *
Exocelina
*, Indonesia, Papua, systematics, taxonomy

## Abstract

Three new species of the genus *Exocelina* Broun, 1886: *E. brazza***sp. nov.**, *E. amabilis***sp. nov.** and *E. mimika***sp. nov.** are described from New Guinea. The former two species are placed into the *E. ekari* group, while the latter is suggested to be a member of a separate lineage, the newly introduced *E. skalei* group. The only other species of that group is *E. skalei* Shaverdo & Balke, 2014. The group is phylogenetically sister of the *E. ekari* group. All species have been collected on the southern slopes of the New Guinea central range, a region that remains mostly unsampled.

## Introduction

Three new species of the genus *Exocelina* Broun, 1886 were discovered on the southern slopes of the New Guinea central range. Two of them, *E. brazza* sp. nov. and *E. amabilis* sp. nov., belong to the largest *Exocelina* species group, the *E. ekari* group. To date, this group contains 56 species (including the two new species) endemic to New Guinea (Balke 1998; Shaverdo et al. 2005, 2012, 2014, 2016, 2020; Shaverdo and Balke 2019). The third species, *E. mimika* sp. nov., shares the same combination of characters including reduced setation of the paramere with *E. skalei* Shaverdo & Balke, 2014. For these two species, the *E. skalei* group, which is the sister group of the *E. ekari* group, is proposed. *Exocelina skalei* is only known from Kaimana in the Bird’s Neck region of New Guinea, which is geographically in principle a westward extension of the central highlands, with Kaimana situated at the south coast.

Including the results of this work, 145 species of *Exocelina* are now described from New Guinea and 202 species worldwide (Nilsson and Hájek 2020; Shaverdo et al. 2020). As in most of our previous papers on the genus, all species data will be presented on the species-id.net portal automatically created by ZooKeys with the publication of this paper.

## Materials and methods

The present work is based on material from the following collections:

**KSP** Koleksi Serangga Papua, at the Biology Department of Universitas Cenderawasih (UNCEN), Waena, Papua, Indonesia;

**MZB**Museum Zoologicum Bogoriense, Cibinong, Indonesia.

Our methods follow those described in detail in our previous articles (Shaverdo et al. 2012, 2014; Shaverdo and Balke 2014). The terminology to denote the orientation of the genitalia follows Miller and Nilsson (2003). All specimen data are quoted as they appear on the labels attached to the specimens. Label text is cited using quotation marks; comments in square brackets are ours. The following abbreviations were used: TL (total body length), TL-H (total body length without head), MW (maximum body width).

## Species descriptions and taxonomy notes

### Exocelina brazza

Taxon classificationAnimaliaColeopteraDytiscidae

Shaverdo & Balke sp. nov.

2E0FD411-D38F-5CDC-8395-2CAE3D1FB329

http://zoobank.org/5114E4B1-7F5D-4ED4-B74A-6B4A485FDF55

**Figure 1.**
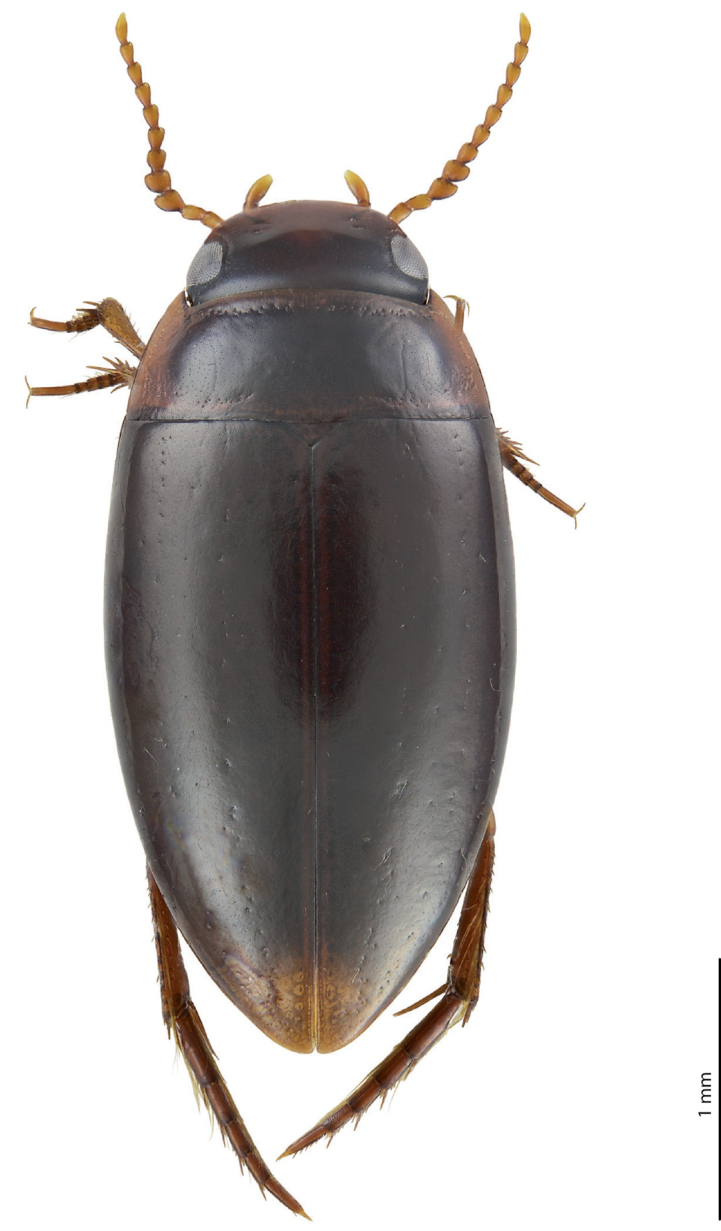
Habitus and colouration of *Exocelina brazza* sp. nov., holotype.

**Figure 2.**
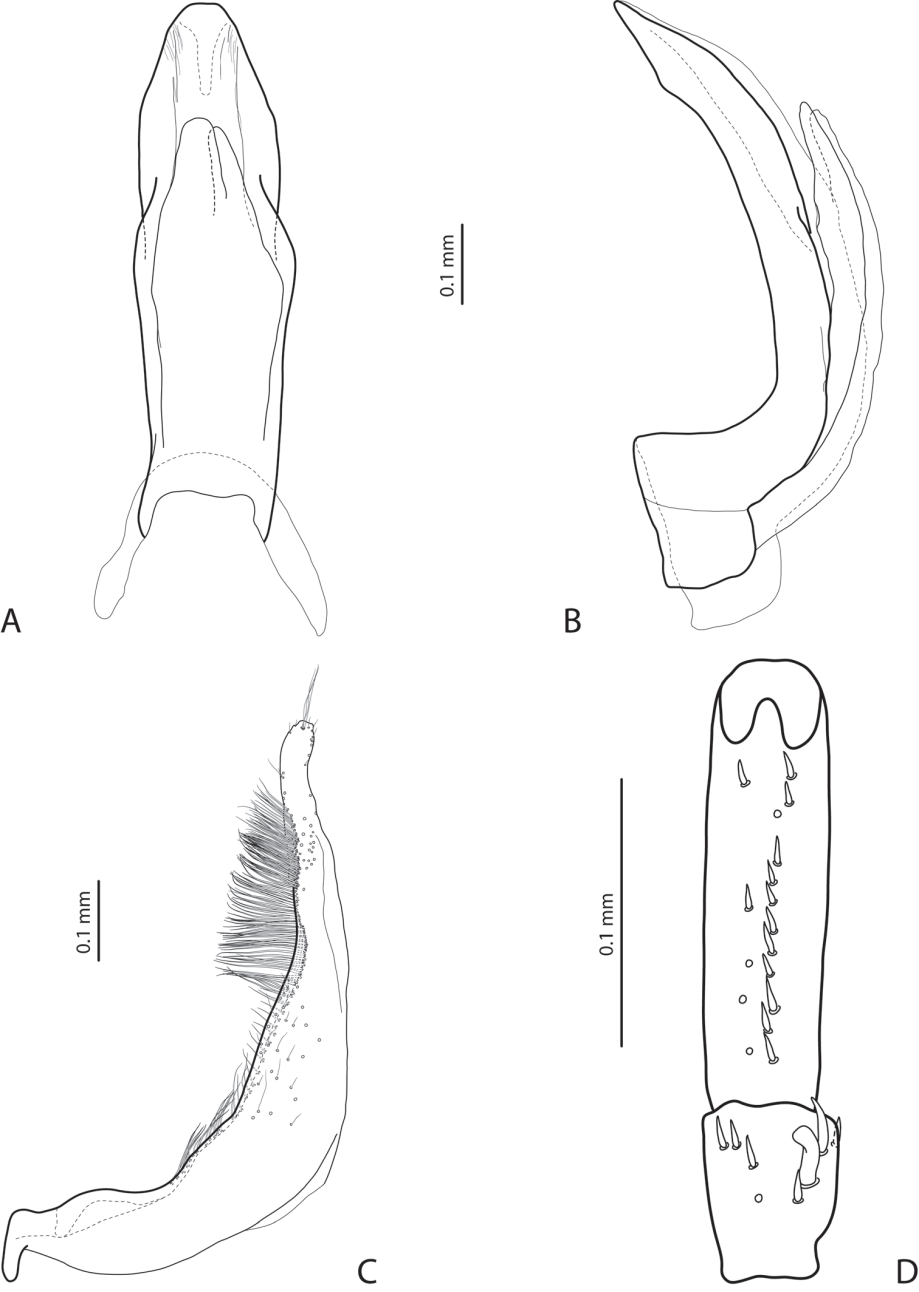
*Exocelina brazza* sp. nov., paratype **A** median lobe in ventral view **B** median lobe in lateral view **C** right paramere in external view **D** right male protarsomeres 4–5 in ventral view.

**Figure 3.**
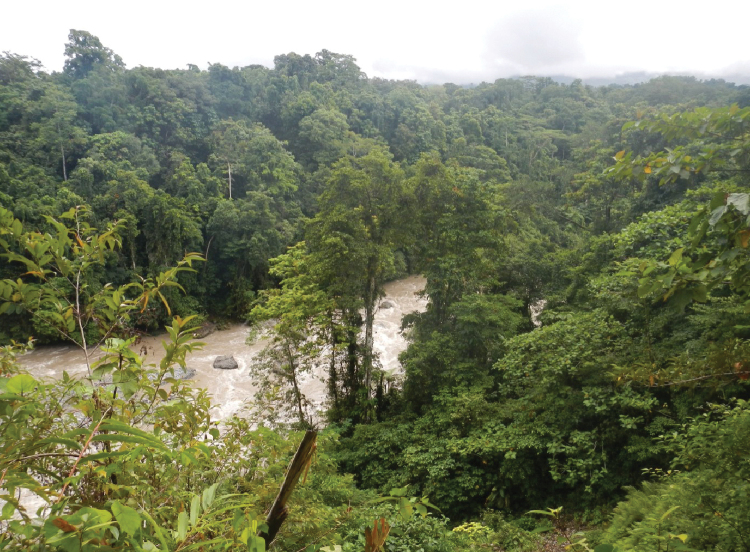
Habitat area of *Exocelina brazza* sp. nov., a view from the forest down into Brazza River.

**Figure 4.**
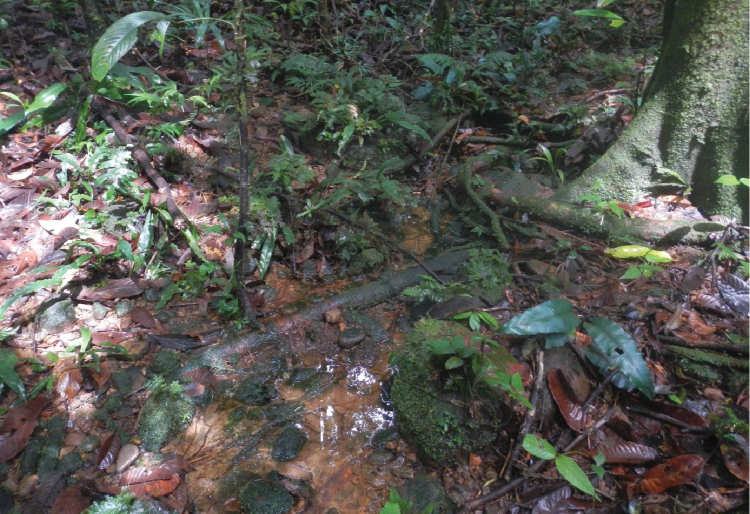
Habitat of *Exocelina brazza* sp. nov., a small creek in the primary forest, ca 100 m from the Brazza River.

**Figure 5.**
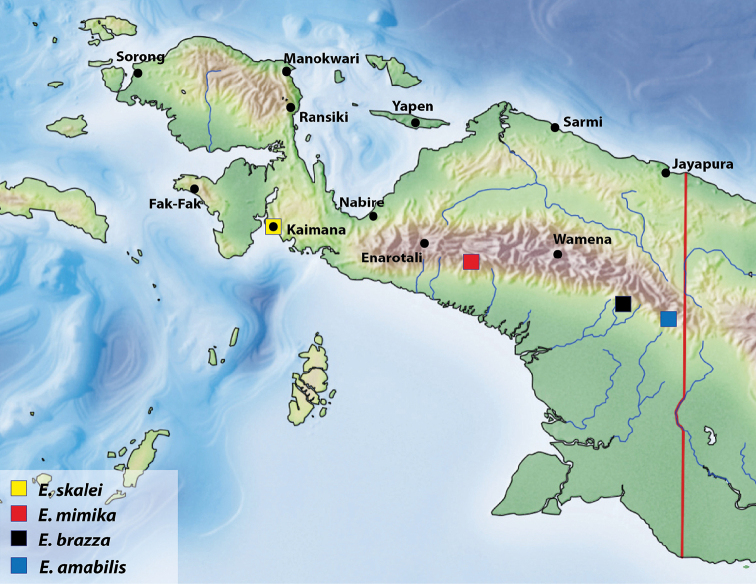
Map of the western part of New Guinea showing the species distribution.

Figs 1 , 2 , 3 , 4 , 5

#### Type locality.

Indonesia: Papua Province, Yahukimo Regency, Dekai, upper Brazza River, near 04°44'27.9"S, 139°39'15.2"E, 300 m a.s.l.

#### Type material.

***Holotype***: male “Indonesia: Papua, Dekai, upper Brazza, 300 m, 2/3.vi.2015, near -4,741084724 139,654211075976, Sumoked (Pap045)” (MZB). ***Paratypes***: 2 males, 10 females with the same label as the holotype, one male and one female additionally with green text labels “6991” and “6990”, respectively (KSP, MZB).

#### Description.

***Body size and form***: Beetle small: TL-H 3.05–3.3 mm, TL 3.45–3.7 mm, MW 1.65–1.8 mm (holotype: TL-H 3.3 mm, TL 3.7 mm, MW 1.8 mm), with oblong-oval habitus (Fig. 1).

***Colouration***: Dorsally brown, with reddish pronotal sides (Fig. 1). Head reddish in anterior half (in front of eyes) and reddish-brown to brown in posterior half (at eye level and behind); pronotum reddish-brown on disc and with broad reddish sides, sometimes also reddish along anterior and posterior margins; elytron reddish-brown to dark brown, with reddish sutural lines; head appendages yellow to yellowish-red, legs reddish. Teneral specimens paler.

***Surface sculpture***: Shiny dorsally, with fine punctation and microreticulation. Head with dense punctation (spaces between punctures 1–3 times size of punctures), distinctly finer and sparser anteriorly and posteriorly; diameter of punctures smaller than diameter of cells of microreticulation. Pronotum with distinctly finer and relatively sparser punctation than on head. Elytra with very sparse and fine punctation, almost invisible. Pronotum and elytra with weakly impressed microreticulation; head with stronger microreticulation. Metaventrite and metacoxa distinctly but weakly microreticulate, metacoxal plates with longitudinal strioles and transverse wrinkles. Abdominal ventrites with weak microreticulation, strioles, and fine sparse punctation, coarser and denser on two last abdominal ventrites.

***Structures***: Pronotum with distinct lateral bead. Base of prosternum and neck of prosternal process with ridge, slightly rounded anteriorly. Blade of prosternal process broadly lanceolate, relatively broad and short, slightly convex medially, with distinct bead and few setae. Abdominal ventrite 6 broadly rounded apically.

***Male***: Antenna modified: antennomeres 3–4 strongly enlarged, antennomere 5 distinctly enlarged and antennomeres 6–10 stout (Fig. 1). Pro- and mesotarsomeres 1–3 not dilated. Protarsomere 4 cylindrical, narrow, with large, thick, strongly curved anterolateral hook-like seta. Protarsomere 5 ventrally with anterior row of 12 and posterior row of 5 short setae (Fig. 2D). Median lobe with discontinuous outline and distinct submedian constriction in ventral view; apex elongate in lateral view (Fig. 2A, B). Paramere without distinct dorsal notch; subdistal part with relatively long, dense setae; proximal setae inconspicuous (Fig. 2C). Abdominal ventrite 6 broadly rounded, with 10–14 lateral striae on each side.

***Female***: Pro- and mesotarsi not modified. Abdominal ventrite 6 without striae.

#### Habitat.

The specimens were collected from a small creek on an almost flat primary forest floor, about 100 m from the upper Brazza River (Figs 3, 4).

#### Distribution.

Indonesia: Papua Province. This species is known only from the type locality (Fig. 5).

#### Etymology.

The species is named after the Brazza River. The name is a noun in the nominative singular standing in apposition.

#### Affinities.

The species evidently belongs to the *E. ekari* group due to the discontinuous outline of its median lobe. Within the group, it can be placed close to the shiny species with antennomeres 3 and 4 larger than other antennomeres (including the recently described *E. athesphatos*Shaverdo et al., 2020 and *E. tsinga*Shaverdo et al., 2020), to which it is assumed to be closely related. However, *E. brazza* sp. nov. can be easily distinguished from all of them by its small size, shape of the male antennae, median lobe and paramere.

### Exocelina amabilis

Taxon classificationAnimaliaColeopteraDytiscidae

Shaverdo & Balke sp. nov.

37E1527A-3691-5673-A58C-B38E09A82CFC

http://zoobank.org/C7F77D9E-27BD-446D-9E9E-415D6B6D143C

Figs 5 , 6 , 7

#### Type locality.

Indonesia: Papua Province, Pegunungan Bintang Regency, south from Ok Sibil, tributary Digul River, 05°03'25.9"S, 140°43'21.1"E, 359 m a.s.l.

#### Type material.

***Holotype***: male “Indonesia: Papua, S Ok Sibil, tributary Digul Riv [River], 359m, 9.vi.2015, -5,05718389 140,722535848617, Sumoked (Pap051)” (MZB). ***Paratypes***: 3 males, 8 females with the same label as the holotype, 2 males additionally with green text label “6997” and “6998” (KSP, MZB).

#### Description.

**Figure 6.**
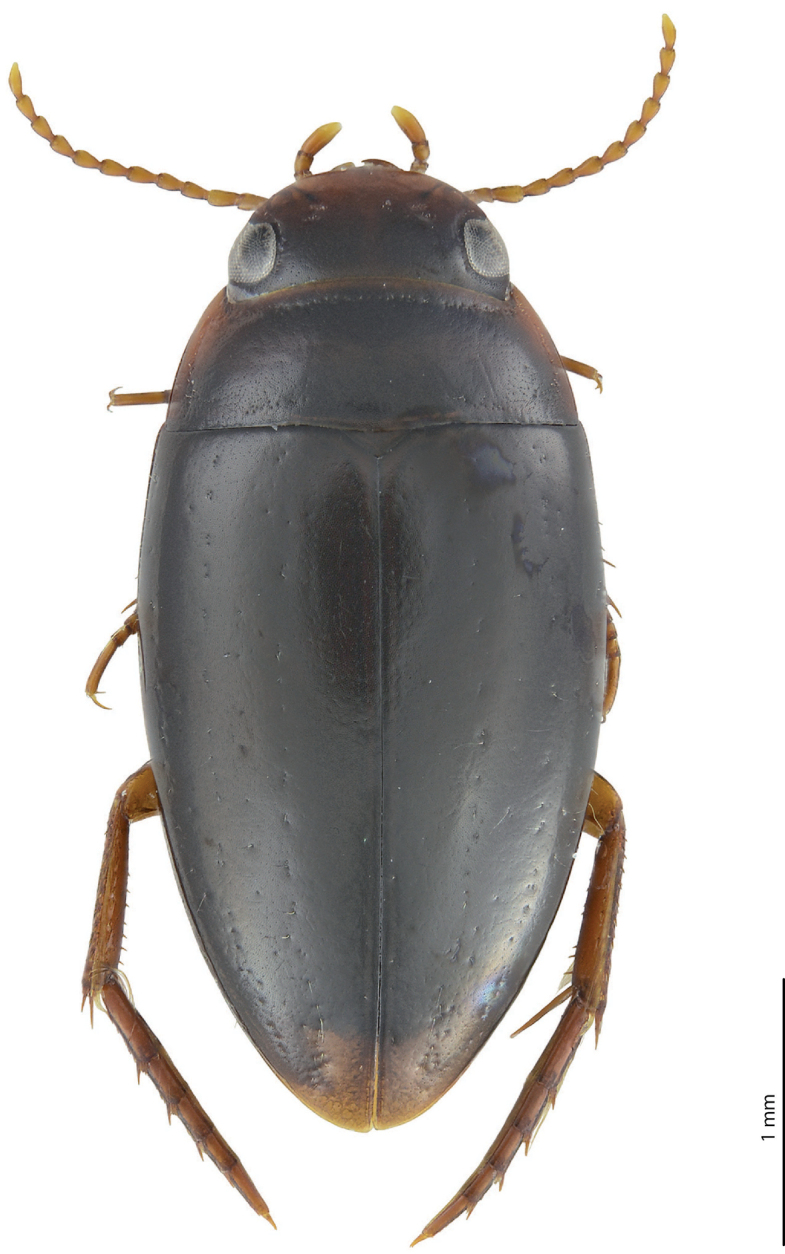
Habitus and colouration of *Exocelina amabilis* sp. nov., holotype.

***Body size and form***: Beetle small: TL-H 2.65–3.45 mm, TL 3.55–3.85 mm, MW 1.75–1.9 mm (holotype: TL-H 3.25 mm, TL 3.65 mm, MW 1.8 mm), with oblong-oval habitus (Fig. 6).

***Colouration***: Dorsally piceous (Fig. 6), with dark brown posterior part of head and lateral parts of pronotum, and sometimes with middle part of pronotum and elytral sutural lines; head appendages and legs yellowish-red, metathoracic legs darker distally.

***Surface sculpture***: Relatively shiny dorsally, with very fine, sparse punctation and distinctly impressed microreticulation. Head with dense punctation (spaces between punctures 1–3 times size of punctures), distinctly finer and sparser anteriorly and posteriorly; diameter of punctures almost equal to diameter of cells of microreticulation. Pronotum with much sparser and finer punctation than head. Elytra with very sparse and fine punctation, often inconspicuous. Pronotum and elytra with distinctly impressed microreticulation; head with microreticulation stronger. Metaventrite and metacoxae distinctly microreticulate, metacoxal plates with longitudinal strioles and transverse wrinkles. Abdominal ventrites with distinct microreticulation, strioles, and very fine and sparse punctation.

***Structures***: Pronotum with narrow lateral bead. Base of prosternum and neck of prosternal process with distinct ridge, slightly rounded anteriorly. Blade of prosternal process lanceolate, relatively broad, slightly convex, with distinct lateral bead and few setae.

**Figure 7.**
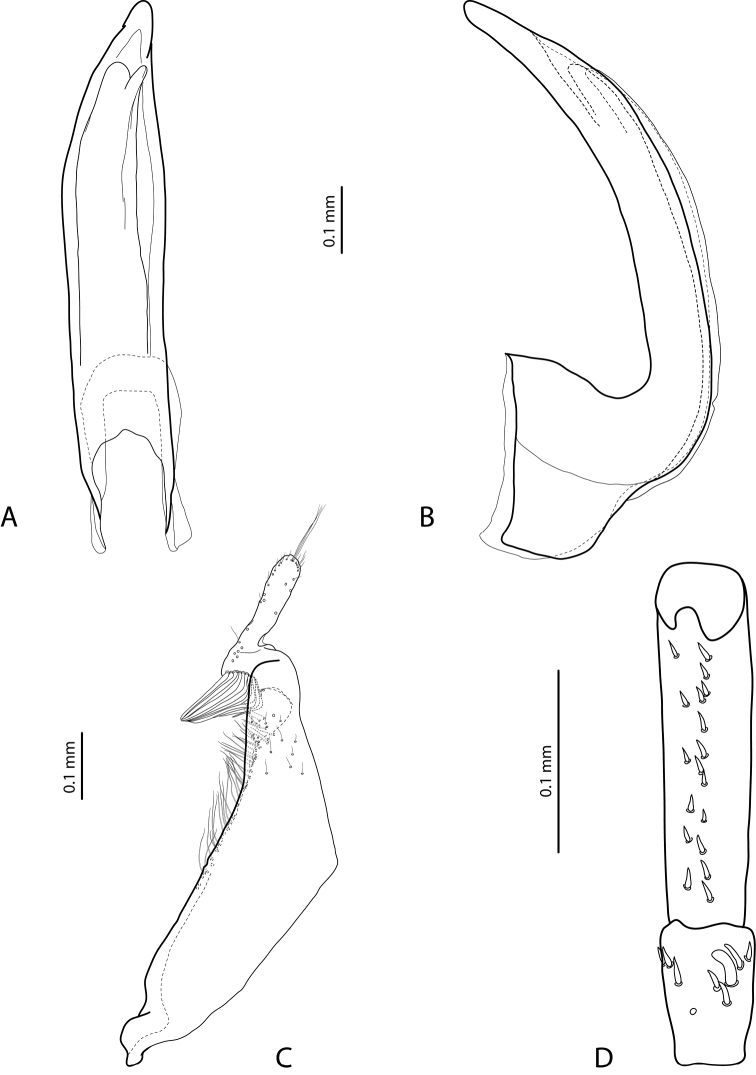
*Exocelina amabilis* sp. nov., paratype **A** median lobe in ventral view **B** median lobe in lateral view **C** right paramere in external view **D** right male protarsomeres 4–5 in ventral view.

***Male***: Antenna simple. Pro- and mesotarsomeres 1–3 not dilated. Protarsomere 4 cylindrical, narrow, with medium-sized, thick, slightly curved anterolateral hook-like seta. Protarsomere 5 ventrally with anterior row of 12 and posterior row of 6 short, thick, pointed setae (Fig. 7D). Median lobe with slightly discontinuous outline (see apically); in lateral view, more or less evenly curved, with broadly pointed apex; in ventral view, tapering to broadly pointed apex (Fig. 7A, B). Paramere with very deep dorsal notch, separating subdistal part; subdistal part broad, transverse, situated under apex of proximal part, with large tuft of dense, thick, flattened setae; proximal setae sparse, thin, much more inconspicuous than subdistal (Fig. 7C). Abdominal ventrite 6 with extremely weak, small medial impression, visible only apically, concave apically, with 12–16 lateral striae on each side.

***Female***: Pro- and mesotarsi not modified. Abdominal ventrite 6 slightly truncate or very slightly concave apically, without medial impression and lateral striae.

#### Habitat.

The specimens were collected from the gravel banks of a primary forest stream. The beetles were mainly hidden in the coarse gravel and were only obtained after some digging.

#### Distribution.

Indonesia: Papua Province. The species is known only from the type locality (Fig. 5).

#### Etymology.

The species name is a Latin adjective and means “loveable”.

#### Affinities.

The species evidently belongs to the *E. ekari* group due to the discontinuous outline of its median lobe. It is similar to *E. utowaensis* Shaverdo, Hendrich & Balke, 2012 in modification of the abdominal ventrite 6, body size, colouration and shape but distinctly differs from it in having a pronotal bead (absent in *E. utowaensis*) and different shape of the median lobe, paramere, and anterolateral hook-like seta of the male protarsomere 4 (it is also larger in *E. utowaensis*). The species is also similar to *E. athesphatos* in modification of the abdominal ventrite 6 and paramere, but distinctly differs from it in smaller size and having simple male antennae.

### Exocelina mimika

Taxon classificationAnimaliaColeopteraDytiscidae

Shaverdo & Balke sp. nov.

FF858FB3-0C02-5079-9DF8-323697254F96

http://zoobank.org/368BA68B-265B-4FEC-BCF0-080373747401

Figs 5 , 8 , 9

#### Type locality.

Indonesia: Papua Province, Mimika Regency, Tsinga Village, Beanekogom River, 04°11.629'S, 137°13.756'E, 1690 m a.s.l.

#### Type material.

***Holotype***: male “Indonesia: Papua, Kabupaten [Regency] Mimika, Desa [Village] Tsinga, Sungai [River] Beanekogom,”, “1690m, 25–30.v.2017, 04°11.629'S, 137°13.756'E, B. Sumoked (Pap66-Bob04)” (MZB). ***Paratypes***: 3 males with the same label as the holotype (KSP, MZB).

#### Description.

**Figure 8.**
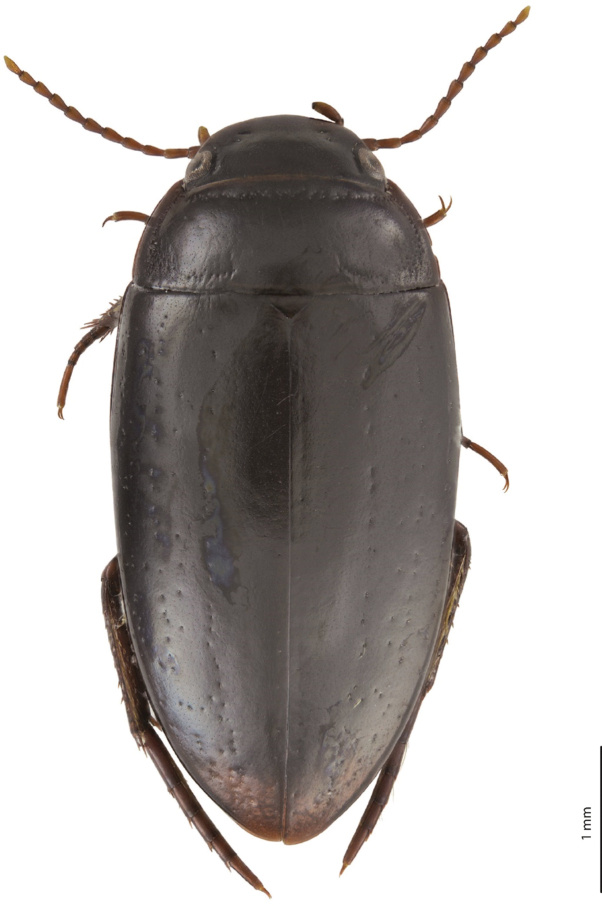
Habitus and colouration of *Exocelina mimika* sp. nov., holotype.

***Body size and form***: Beetle medium-sized: TL-H 4.8–5.0 mm, TL 5.2–5.4 mm, MW 2.5–2.6 mm (holotype: TL-H 4.8 mm, TL 5 mm, MW 2.5 mm), with oblong-oval habitus (Fig. 8).

***Colouration***: Dorsally piceous (Fig. 8), sometimes with dark brown posterior part of head, lateral sides of pronotum, and dark brown elytral sutural lines; head appendages yellowish brown, legs yellowish brown to reddish-brown. Teneral specimens paler.

***Surface sculpture***: Shiny dorsally, with distinct punctation and weakly impressed microreticulation. Head with relatively dense, unevenly distributed punctation (spaces between punctures 1–4 times size of punctures), distinctly finer and sparser anteriorly and posteriorly; diameter of punctures equal to diameter of cells of microreticulation. Pronotum and elytra with punctation sparser and finer than on head and with distinct but weakly impressed microreticulation; head with stronger microreticulation. Metaventrite and metacoxae distinctly microreticulate, metacoxal plates with longitudinal strioles and transverse wrinkles. Abdominal ventrites with distinct microreticulation, strioles, and fine but distinct punctation.

***Structures***: Pronotum with distinct lateral bead. Base of prosternum and neck of prosternal process with distinct ridge, very slightly rounded anteriorly. Blade of prosternal process lanceolate, narrow, slightly convex, with distinct lateral bead and few setae. Abdominal ventrite 6 broadly rounded.

**Figure 9.**
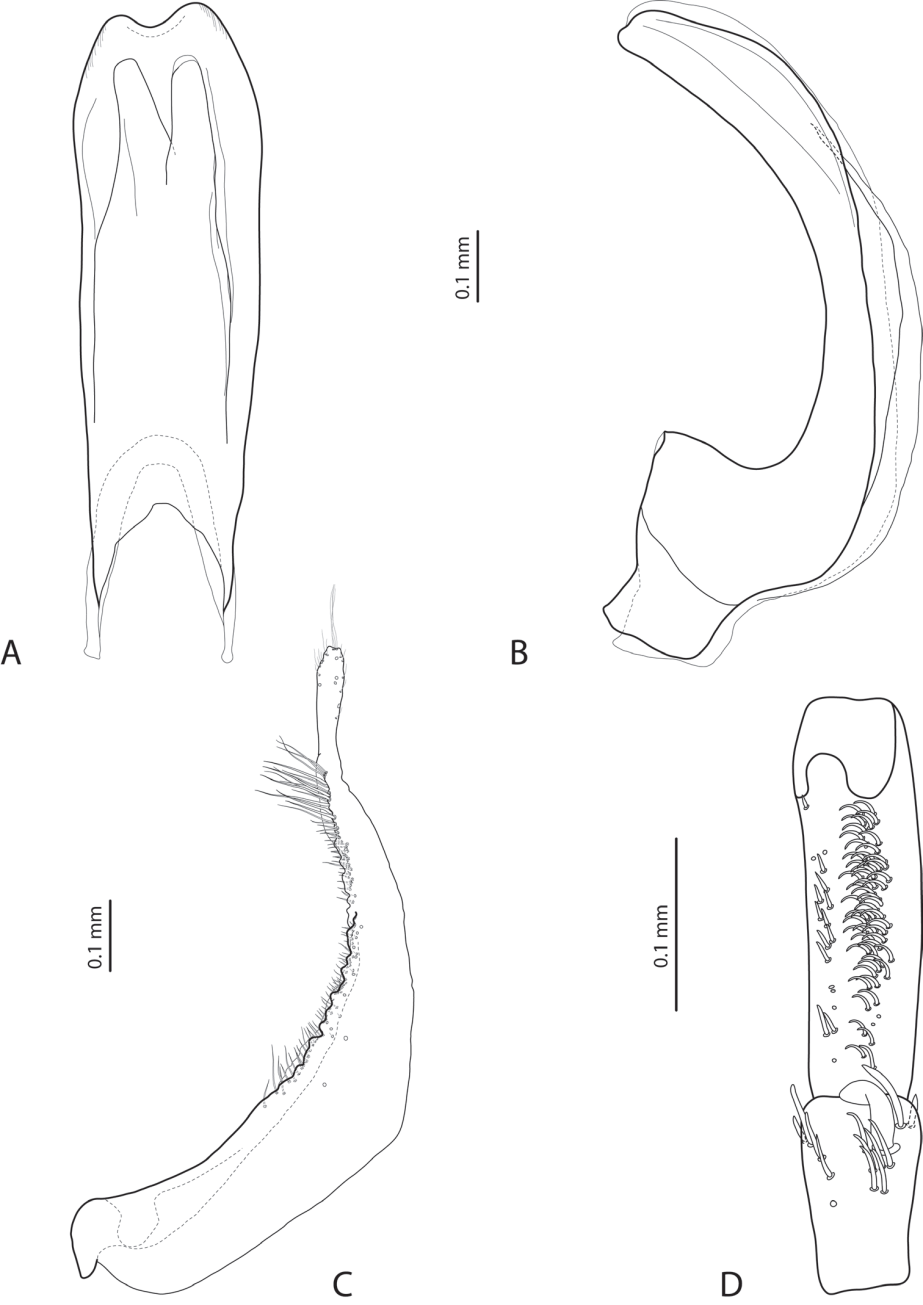
*Exocelina mimika* sp. nov., paratype **A** median lobe in ventral view **B** median lobe in lateral view **C** right paramere in external view **D** right male protarsomeres 4–5 in ventral view.

***Male***: Antenna simple. Pro- and mesotarsomeres 1–3 very slightly dilated. Protarsomere 4 simple, narrow, with large, thick, strongly curved anterolateral hook-like seta. Protarsomere 5 ventrally with anterior band of more than 50 and posterior row of 20 relatively long setae (Fig. 9D). Median lobe robust, with continuous outline and without setation; lateral margins thick in apical half; apex with very short blunt prolongation in lateral view and deeply concave in ventral view (Fig. 9A, B). Paramere without dorsal notch and with reduced setation: setae very thin, inconspicuous, sparse, longer subdistally and proximally and very short medially (Fig. 9C). Abdominal ventrite 6 broadly rounded, with 2–7 lateral striae on each side.

***Female***: Unknown.

#### Habitat.

The specimens were collected from rock pools at the edge of a fast-flowing mountain stream.

#### Distribution.

Indonesia: Papua Province. The species is known only from the type locality (Fig. 5).

#### Etymology.

The species is named after Mimika Regency, where it was collected. The name is a noun in the nominative singular standing in apposition.

#### Affinities and introduction of the *E. skalei* group.

The other *Exocelina* species known from the Tsinga area is *E. tsinga*Shaverdo et al., 2020, which is very similar to the new species in size, body shape, colouration, and surface sculpture. The males of these species can be easily distinguished due to the modified antennae of *E. tsinga* and different shape and setation of their genitalia; female identification, however, could be problematic.

Based on morphological characters, we cannot place the new species in any known species group. The new species resembles representatives of the *E. jaseminae* group in the shape of the median lobe and *E. mekilensis* Shaverdo & Balke, 2019 and species of the *E. ullrichi* group (Shaverdo and Balke 2014) in the reduced setation of the paramere. However, in the shape of the median lobe and setation of the paramere, the new species is the most similar to *E. skalei*. Thus, we assume that these two species might be closely related. *Exocelina skalei* has been so far treated as a member of the *E. ekari* group but according to the molecular analyses, it is the sister clade of the *E. ekari* group (Toussaint et al. 2014, 2015).

Therefore, for *E. skalei* and *E. mimika* sp. nov., herein we introduce a new species group, the *E. skalei* group, with the following diagnostic characters:

- beetle small or medium-sized (TL-H 2.9–5.0 mm), with continuous body outline of broadly oval or oblong-oval shape;
- elytra without striae/strioles, with distinct punctation, beetles shiny or submatt;
- pronotum with distinct lateral bead;
- antennomeres not modified;
- male protarsomere 4 simple, with medium-sized or large, slightly or strongly curved anterolateral hook-like seta;
- median lobe of aedeagus without setation, with continuous or slightly discontinuous apically outline;
- paramere without dorsal notch and with strongly reduced setation: setae very thin and sparse, some longer setae distinct subdistally; setae tiny, inconspicuous medially and proximally.

Since the southern slopes of the central range is a poorly studied area, we assume that more species of the *E. skalei* group await discovery. The new material, including its molecular analysis, would help to confirm group delimitation.

## Supplementary Material

XML Treatment for Exocelina brazza

XML Treatment for Exocelina amabilis

XML Treatment for Exocelina mimika
